# Hes1 in malignant tumors: from molecular mechanism to therapeutic potential

**DOI:** 10.3389/fimmu.2025.1585624

**Published:** 2025-07-18

**Authors:** Liping Zhang, Qian Zhang, Cheng Guo, Zixin Ru, Zetian Yang, Yi Geng, Junjie Yang, Daigui Zhang, Zhenhuai Yang, Shuicai Huang

**Affiliations:** The Affiliated Guangzhou Hospital of TCM of Guangzhou University of Chinese Medicine, Guangzhou, Guangdong, China

**Keywords:** Hes1, malignant tumors, signal transduction, therapeutic targets, tumor microenvironment

## Abstract

The occurrence and development of malignant tumors involve abnormalities in complex molecular regulatory networks, among which the abnormal activation of the transcriptional regulator hairy and enhancer of split 1 (Hes1) has attracted significant attention in recent years and is closely associated with prognosis in various malignancies. Hes1 exhibits high expression in various solid tumors and hematological malignancies, where it participates in alterations involving diverse immune cells, inflammatory factors, and the immune microenvironment, thereby promoting tumor cell proliferation, invasion, metastasis, and resistance to treatment. Recent studies have widely investigated the potential of targeting Hes1 and inhibiting its expression as a cancer therapeutic strategy, although its precise mechanisms of action are not yet fully elucidated. Hes1 interacts with critical pathways including Notch, JAK/STAT, PI3K/AKT/mTOR, and Wnt/β-catenin. These interactions form complex crosstalk networks that drive malignant transformation and progression. Furthermore, Hes1 plays a central role in the formation of an immunosuppressive tumor microenvironment (TME) and immune escape by regulating the expression of immune checkpoint-associated proteins, extracellular matrix (ECM) remodeling, and other processes, making it a highly promising therapeutic target. Notably, the expression level of Hes1 is significantly correlated with tumor clinical stage, prognosis, and drug resistance. This review comprehensively introduces the mechanisms of Hes1 in the progression of malignant tumors, with a particular focus on discussing its application and underlying mechanisms in tumor immunotherapy. It integrates the latest clinical evidence and preclinical research perspectives. The goal is to highlight the translational potential of Hes1 as a novel biomarker and molecular target.

## 1 Introduction

Hes1 is a protein-coding gene that belongs to the basic helix-loop-helix (bHLH) family. Hes1 was first discovered in Drosophila, and subsequently found to be widely present in mammalian cells (1, 2). The Hes1 protein, encoded by the Hes1 gene, contains 280 amino acids with a molecular weight of approximately 29.4 kDa (3, 4). Hes1 plays a crucial role in the development of mammals and in various physiological processes, including the regulation of the cell cycle, control of cell proliferation and differentiation, and the maintenance of stem/progenitor cells (5–7). However, increasing evidence demonstrates that Hes1 is aberrantly expressed in many human malignancies Hes1 can serve as a potential prognostic biomarker for cancer and is expected to become a significant target in the field of cancer therapy. Hes1 is closely associated with tumor immunotherapy. Its aberrant expression correlates significantly with the immune phenotype of malignancies, immune checkpoint activation, immune cell recruitment, TME remodeling, and treatment prognosis. The expression and activity of Hes1 in cancer are primarily regulated by the evolutionarily conserved canonical Notch pathway, and are also influenced by oncogenes, epigenetics, the microenvironment, and various other signaling pathways. The role and prognostic value of Hes1 in different cancers remain controversial, and its specific functions and regulatory mechanisms still require further investigation to ensure the effectiveness of targeting strategies. Therefore, this article summarizes the functions and related molecular mechanisms of Hes1, discusses its important role in tumor immunity, and addresses the strategies for targeting Hes1 and the potential challenges, providing insightful information and evidence for future cancer therapies targeting Hes1.

## 2 Structure of Hes1 and its regulatory mechanisms

### 2.1 Molecular characterization of the Hes1 protein

**Figure 1:**
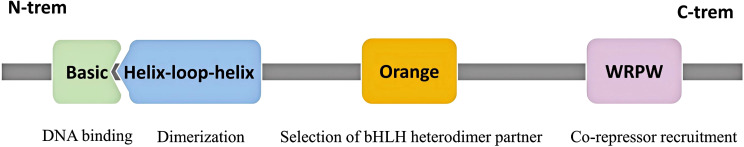
Schematic of Hes1 protein with its domains and corresponding functions. The Hes1 protein consists of three conserved domains: the bHLH domain, the Orange domain, and the WRPW motif. The bHLH domain, located at the N-terminus, comprises the Basic (b) region and the Helix-loop-helix (HLH) region. The Basic region mediates DNA binding, while the HLH region is involved in dimerization. The Orange domain specifically selects binding partners and recruits co-repressors. The WRPW motif, located at the C-terminus, enhances transcriptional repressive activity by binding to co-repressors.

The bHLH proteins, named for the highly conserved bHLH motif within their protein structure, are a class of transcription factors present in eukaryotes, and they constitute a large family, known as the bHLH superfamily, which controls many aspects of eukaryotic development and function (8–10).The Hes family belongs to the bHLH transcription factor superfamily and currently comprises seven protein members (Hes1-7). These members exhibit homology at the amino acid level in the bHLH domain and regulate cell proliferation, differentiation, and stem cell maintenance, playing a significant role particularly in the physiological and pathological processes of the nervous system (7, 11, 12). Hes1 primarily functions as a negative regulatory transcription factor and is crucial for the development and differentiation of various cell types. Hes1 contains three evolutionarily conserved domains: the bHLH domain, he Orange domain, and the WRPW domain (13) (**Figure 1**).

The bHLH domain consists of two main parts: the basic region and the HLH region. The N-terminal basic region can recognize and bind to specific DNA sequences, while the C-terminal HLH region facilitates dimerization with other Hes proteins or accessory proteins. Transcription factors containing the bHLH structure typically bind to the E-box or its variant sequences to regulate the transcriptional activity of downstream genes, whereas Hes factors exhibit higher affinity for N-box (CACNAG) sequences or Class C sites (CACGCG), and this unique DNA-binding activity is believed to be conferred by the proline residues in the basic region. In summary, the bHLH domain enables Hes1 to form homodimeric or heterodimeric complexes that bind to DNA targets, thereby regulating the expression of target genes (1, 2, 14, 15). The Orange domain, located downstream of the bHLH domain, regulates the selection of bHLH heterodimer partners and mediates protein-protein interactions (7, 16, 17). The C-terminal WRPW domain contains the highly conserved tetrapeptide sequence Trp-Arg-Pro-Trp (WRPW). This sequence interacts with the co-repressors encoded by the transducin-like enhancer/Groucho-related gene (TLE/GRG), thereby enhancing the transcriptional repressive capability of the Hes1 protein (18, 19).

### 2.2 The inhibitory and activating effects of Hes1

**Figure 2:**
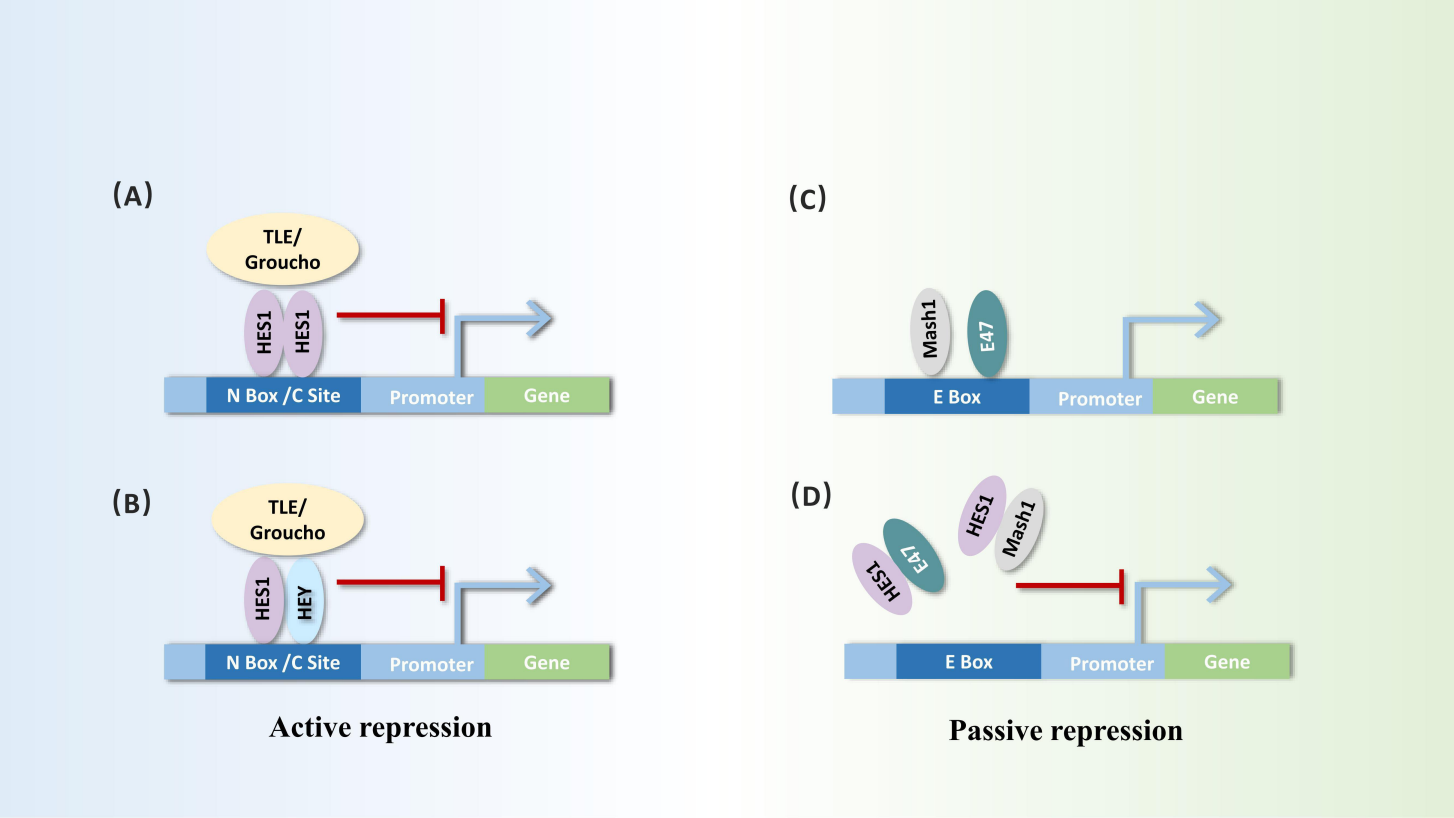
Dual repressor functions of Hes1: active repression and passive repression. Active repression process: **(A, B)** Hes1 forms homodimers or heterodimers with itself or other bHLH factors such as Hey at the N-box or C-type sites of target gene promoters. It initiates active repression through interaction with corepressors, such as TLE/Groucho homologs. Passive repression process: **(C, D)** Hes1 can bind to bHLH activators (e.g., Mash1 and E47), inhibiting their ability to bind to the E-box of target genes, thereby triggering passive repression.

Hes1 is widely recognized as a transcriptional repressor, whose transcriptional repression mechanism can be divided into active and passive inhibition. Hes1 can actively repress transcription by directly binding to the promoter region of target genes. Utilizing its unique WRPW, Hes1 forms a transcriptional repressor complex with the core repressor proteins of the TLE/GRG family, recruiting histone deacetylases (HDACs) to alter chromatin structure, thereby inhibiting gene transcription activity. During active repression, Hes1 can not only form homodimers but also heterodimers with bHLH repressors such as Hey1 or Hey2, binding to the N-box or class C site sequences in the promoter regions of target genes to repress transcription (20, 21). Hes1 can also exert passive inhibitory effects by forming heterodimers with bHLH activators such as Mash1 and E47, preventing bHLH activators from binding to E-box sequences to activate the transcription of downstream genes (2, 22, 23) (**Figure 2**).

In addition, Hes1 can recruit SIRT1, a member of the HDACs family, via the bHLH structural domain, which is involved in histone deacetylation, thereby enhancing the inhibitory effect of Hes1 to repress target genes in a TLE-independent manner (24). Besides the modification of transcription initiation, Hes1 can also inhibit the recruitment of the positive transcription elongation factor b (P-TEFb), preventing phosphorylation of RNA polymerase II (Pol II) at Ser2 and productive elongation, thereby suppressing the rate of transcriptional elongation of target genes and inhibiting transcription (25).

Hes1 can also function as a transcriptional activator under specific conditions. It has been reported that Hes1 can directly bind to STAT3 and induce its phosphorylation and activation by recruiting JAK2 (26). Additionally, Hes1 can cooperate with pRb to activate RUNX2-dependent transcription (27). Furthermore, in triple-negative breast cancer (TNBC), Hes1 promotes the transcription of the Slug by directly acting on its promoter (28). The transcriptional activation function of Hes1 may be influenced by its interactions with other factors, transcriptional complexes formed, and cell type-specific factors (29). Therefore, further investigation into the dual regulatory roles of Hes1 will contribute to understanding its functions in cell biology and disease, potentially providing new perspectives and ideas for the development of related therapeutic strategies.

### 2.3 The upstream regulatory network of Hes1

**Figure 3:**
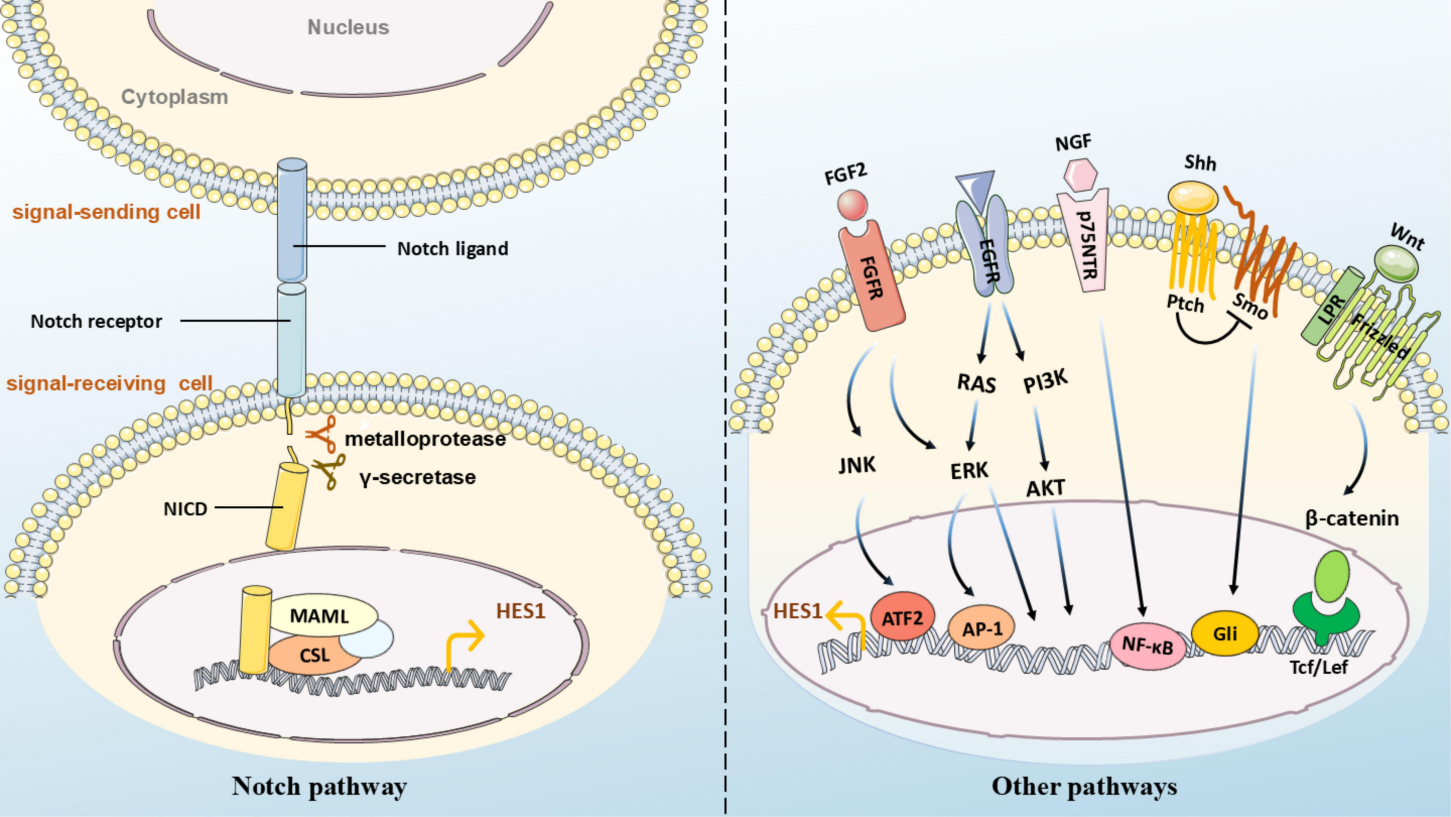
(Left) Notch pathway:  Notch ligands (e.g., Delta/Jagged) on adjacent cells bind to receptors on the target cell membrane. Cleavage by ADAM proteases and γ-secretase releases NICD, which translocates to the nucleus, binds CSL with co-activators (e.g., MAML), and initiates transcription of hes1.  (Right) Other pathways: - Wnt: Binds Frizzled/LRP5/6 to activate β-catenin nuclear translocation and hes1 transcription via Tcf/Lef; - Hedgehog (Hh): Binds Ptch to release Smo inhibition, activating Gli for direct hes1 promoter binding; - FGF2: Recruits ATF2 to the Hes1 promoter; - NGF: Binds p75NTR to upregulate Hes1  via NF-κB; - EGF: Modulates  Hes1 through ERK/AKT cascades.

Hes1 is a crucial downstream effector of the Notch signaling pathway, and its expression and function are highly dependent on the regulation of the Notch signaling pathway (30–32) (**Figure 3**). When Notch ligands such as Delta-like or Jagged, expressed by the signal-sending cells bind to the Notch receptors (Notch1-4) expressed by the signal-receiving cells, the Notch receptors activate. The Notch intracellular domain (NICD) undergoes sequential proteolytic cleavage mediated by metalloproteases and γ-secretase, and is subsequently released, translocating into the nucleus, where it binds to the transcription factor CBF1/Su(H)/Lag-1 (CSL) to form a complex. In the absence of Notch signaling, CSL suppresses the transcription of target genes (33). However, the binding of NICD alters the function of CSL. NICD-CSL forms a transcriptionally active complex by recruiting co-activators, such as members of the MAML family, and displacing co-repressors, thereby activating the Hes1 promoter and promoting Hes1 transcription. Additionally, under hypoxic conditions, when Notch is activated, HIF-1α can be recruited as a Notch-responsive promoter, interacting with NICD and potentially serving as part of the NICD-CSL transcriptional complex, enhancing the expression of direct downstream genes of Notch (34). The Notch-Hes1 signaling axis is involved in regulating cell fate decisions, proliferation, differentiation, and apoptosis, and is closely related to the development and homeostasis of various tissues and organs (35–37).

Hes1 can also be activated by non-Notch pathways. For instance, when Wnt proteins bind to cell-surface receptors Frizzled and LRP5/6, accumulated β-catenin transfers to the cell nucleus and binds to the conserved lef/Tcf binding sites in the promoter region of the Hes1 gene, initiating Hes1 transcription (38). Hes1 has also been identified as a novel downstream bHLH transcription modifier of Sonic Hedgehog (Shh)/Gli signaling (39). The induction of Hes1 by Shh may be Gli-dependent (40) and may require Smo function (41). The upregulation of Hes1 mediated by the Hedgehog signaling pathway promotes the maintenance of an undifferentiated state in ventricular zone stem cells/glial cells (42). Blocking the Hedgehog signaling pathway significantly reduces the expression of Hes1 and Gli2 (43). In the absence of CSL activation, Gli2 in retinal progenitor cells can directly bind to the Hes1 promoter, enhancing Hes1 expression (44). The Gli inhibitor GANT61 suppresses the expression of Hes1, Notch1, and Jagged in the Notch pathway in a dose- and time-dependent manner, thereby inhibiting multiple myeloma cell proliferation and promoting apoptosis (45). In the poorly differentiated endometrial cancer cell line HEC-50B, an autocrine FGF/FGFR signaling stimulates Hes1 expression and cell proliferation (46). FGF induces oscillatory expression of Hes1 through ERK activation, and the oscillation of ERK activity fine-tunes Hes1 oscillation (47). In neuroblastoma cells, Hes1 levels increase in a dose-dependent manner under TGF-α stimulation. TGF-α induces ERK1/2 phosphorylation, activates the Hes1 promoter, thereby increasing Hes1 expression. However, in the absence of TGF-α, Hes1 expression is maintained by phosphorylated ERK1/2, independent of EGFR activation (48). TGF-β, a crucial cytokine that induces epithelial-mesenchymal transition (EMT), can upregulate Hes1 expression in epithelial ovarian cancer (49). In hepatic stellate HSC-T6 cells, TGF-β-BMP signaling can interact with Notch1 to regulate Hes1 expression (50). In hippocampal pyramidal neurons, the neurotrophic factor NGF binds to the neurotrophin receptor p75NTR. This interaction leads to the intracellular accumulation of NF-κB, which subsequently upregulates Hes1 (51, 52). In neural progenitor cells, FGF2 (Fibroblast Growth Factor 2) activates Hes1 expression on the Hes1 promoter by directly binding to the downstream target ATF2 of JNK (53). In confluent growth-arrested endothelial cells, Hes1 expression is also partly mediated by the JNK signaling pathway (54). In colon adenocarcinoma, the upregulation of Hes1 is positively correlated with the transcription factor ETV4, which binds to the Hes1 promoter sequence and activates its transcription (55).

Besides the regulation by upstream signaling pathways and other factors, Hes1 expression is also modulated by its own negative feedback loop. Following the activation of Hes1 transcription by the Notch signaling pathway, Hes1 can directly bind to the DNA sequence of its own promoter, thereby inhibiting transcription. This negative feedback mechanism on its own transcription results in a periodic oscillation pattern of Hes1 (56). Hes1 plays a pivotal role in the proliferation and differentiation of stem cells, especially neural stem cells, through its expression pattern (5). Hes1 regulates the oscillatory and sustained expression of the proneural factor Ascl1. Hes1 oscillation drives the oscillatory expression of Ascl1 through periodic inhibition, activating the proliferation of neural progenitor cells. Conversely, when Hes1 expression disappears, Ascl1 expression persists, promoting neuronal differentiation (57). Recent studies have revealed that Hes1 regulates p12 expression by altering its expression pattern to oscillatory or continuously high expression, thereby activating or inhibiting the proliferation of neural stem cells (58). In ER (58) breast cancer cells, enforcing sustained expression of Hes1, which diminishes its oscillations, results in a slowed cell cycle, impaired proliferation, and abolishment of the dynamic expression of p21 (59).

The stability of Hes1 expression is also influenced by modifications such as phosphorylation and ubiquitination. For instance, Hes1 protein can be rapidly degraded by the ubiquitin-proteasome system (56). Additionally, human cytomegalovirus IE1 has been found to assemble ubiquitinated complexes and promote the ubiquitination of Hes1 (60). Furthermore, Hes1 can undergo SUMOylation, which enhances the stability of Hes1 protein and increases its transcriptional repressive activity on the target gene GADD45α, promoting cell survival (61). MLL1, a conserved chromatin-modifying factor, interacts with WD repeat domain 5 (WDR5) and directly regulates Hes1 transcription through H3K4me3 methylation (62).

In summary, the regulation of Hes1 involves multiple factors, including epigenetic modifications, transcriptional factor regulation, and the activation of various signaling pathways. Further investigation into these factors may offer new insights for the treatment of related diseases.

## 3 Hes1 and tumor biology

### 3.1 Dysregulation of cell cycle and apoptosis

Cell cycle disorder is one of the key characteristics of tumorigenesis and development, leading to imbalances in the proliferation and differentiation of tumor cells. Hes1 drives the transition of cells from G1 to S phase by inhibiting the expression of cell cycle inhibitors such as CDKN1A/p21 and CDKN1B/p27 (39, 63, 64). This implies that in the context of high Hes1 expression, the progression of the cell cycle may be accelerated, promoting continuous proliferation of tumor cells and thus fueling tumor development. For example, upon Hes1 silencing, the proportion of MEC1 and HG3 chronic lymphocytic leukemia cells arrested in the G1 phase increased, whereas the proportion in S phase decreased. Conversely, Hes1 overexpression activated the Notch1 signaling pathway, leading to an increase in S phase-arrested cells and inhibition of apoptosis in both cell lines (65). Furthermore, enforced cell cycle arrest can trigger irreversible cellular senescence. Hes1 regulates the cell cycle to inhibit tumor cell aging. In human rhabdomyosarcoma, sustained high levels of Hes1 prevent p21-mediated quiescent fibroblasts from entering prolonged cell cycle arrest-associated senescence, and its inactivation leads to spontaneous tumor cell differentiation (66). In hepatocellular carcinoma, Hes1 suppresses cellular senescence by inhibiting CDKN1C/P57 (67). Additionally, Hes1 directly inhibits the expression of the BBC3 gene (encoding the pro-apoptotic factor PUMA) in T-cell acute lymphoblastic leukemia (T-ALL), thereby suppressing oncogenic stress-induced apoptosis during T-cell transformation (68). Hes1 also promotes cervical cancer cell proliferation and inhibits differentiation via Hash1 downregulation (69), whereas reduced Hes1 mRNA expression induces apoptosis in these cells (70). Therefore, Hes1 promotes tumor cell proliferation by driving the cell cycle progression and enhances tumor cell viability by inhibiting tumor cell senescence and apoptosis.

### 3.2 Regulation of cancer stem cells, EMT, and multimodal therapy resistance

Cancer stem cells (CSCs) represent a subpopulation of cancer cells with stem cell-like properties, including tumor initiation, self-renewal, multilineage differentiation, and plasticity. These cells are pivotal drivers of tumorigenesis, metastasis, and therapeutic resistance (71). In neuroblastoma, the activation dynamics of the Hes1 promoter reveal CSC plasticity, stemness, and heterogeneity (72). Hes1 shows a positive correlation with CSC markers (such as CD133, CD44, SOX2, Nanog, etc.) in colon, gastric, and breast cancers (28, 73, 74). Elevated Hes1 levels may enhance CSC marker expression, reinforcing stemness. Upregulating Hes1 increases CSC numbers, promotes spheroid formation, improves cell survival, and induces tumorigenicity, self-renewal, and chemoresistance (73, 74). Conversely, Hes1 downregulation suppresses spheroid formation, cell invasion, tumor proliferation/migration, and triggers apoptosis (75–78). *In vivo* studies demonstrate that Hes1 knockdown reduces breast CSC-derived tumor size and weight (28), confirming its role in tumor growth inhibition. Furthermore, CSC-microenvironment interactions regulate maintenance and differentiation. Co-culture of human colorectal Caco-2 cells with pericyte-derived myofibroblasts (18Co) significantly elevates CD13 (62) CD44 (62) cells and Hes1 expression. TME-derived IL-6/IL-8 may mediate myofibroblast-induced CSC expansion through Hes1 activation (79). Collectively, Hes1 correlates with CSC marker levels and critically regulates CSC tumorigenicity, differentiation, self-renewal, migration, and drug resistance. These findings provide novel insights for developing CSC-targeted anticancer therapies.

EMT is a biological process through which cells transition from an epithelial to a mesenchymal phenotype. While essential for embryonic development and tissue repair, aberrant reactivation of EMT-associated pathways during cancer progression drives malignant traits including enhanced migration/invasion, elevated cancer stemness, and increased resistance to chemoresistance or immunotherapy (80). Hes1 promotes EMT by modulating EMT-related gene expression. For example, in colon and breast cancers, Hes1 overexpression downregulates epithelial markers (e.g., E-cadherin) and upregulates mesenchymal markers (N-cadherin, vimentin), thereby inducing EMT and accelerating tumor proliferation, invasion, metastasis, and drug resistance (81–83). Conversely, Hes1 inhibition significantly elevates E-cadherin and ZO1 levels while suppressing N-cadherin, vimentin, and Snail, effectively blocking EMT (74). Mechanistically, Hes1 activates Slug to enhance breast cancer stemness via the Hes1/Slug/EMT axis, further linking stemness to therapeutic resistance (28).

Therapeutic resistance in cancer remains a major clinical challenge. In colorectal cancer RKO and HCT8 cells, Hes1 overexpression upregulates ABC transporters (ABCC1, ABCC2, P-gp), reducing intracellular drug uptake/accumulation and inducing 5-Fu chemoresistance (82). Conversely, Hes1 inhibition downregulates chemoresistance-associated proteins (MDR1, ABCG1/2, RAD51) in gastric cancer MKN45 spheroids (74). Radiotherapy resistance is linked to Notch signaling hyperactivation and DNA repair protein overexpression. Blocking the Notch1/Hes1 axis enhances radiosensitivity by suppressing proliferation, exacerbating radiation-induced DNA damage (e.g., DSBs), and impairing DSB repair in colorectal cancer (84). High Hes1 expression also confers resistance to EGFR-TKIs (trametinib, lapatinib) in low-grade serous ovarian cancer, gastric cancer, and lung adenocarcinoma. Conversely, Hes1 knockdown sensitizes tumors to these agents (74, 85, 86). In breast cancer, elevated Hes1 mRNA correlates with tamoxifen (TAM) resistance, advanced N stage, and nipple involvement, suggesting its role in endocrine therapy failure (87). Notably, Hes1 modulates immunotherapy response: its conditional knockout in tumor-associated macrophages enhances cytotoxic T-cell infiltration/activation, suppressing tumor growth (88).

As a key regulatory factor, Hes1 plays a central role in tumorigenesis, metastasis, and drug resistance by promoting CSC stemness, inducing EMT, and modulating diverse drug resistance mechanisms. Additionally, Hes1 regulates the tumor microenvironment and immune cell activity, further influencing tumor progression. Targeting Hes1 represents a promising strategy to overcome tumor recurrence and drug resistance, with profound clinical translational potential.

### 3.3 Key cancer signaling pathways

#### 3.3.1 Notch signaling pathway

The Notch signaling pathway is a highly conserved intercellular communication mechanism that exhibits dual regulatory roles in tumorigenesis. Its oncogenic or tumor-suppressive effects depend on multiple factors including tumor type, specific upstream/downstream components of the pathway, and hypoxic microenvironment (89, 90). As a core downstream effector, Hes1 mediates the oncogenic potential of Notch signaling. Aberrant activation of the Notch-Hes1 axis is strongly associated with malignant phenotypes across various cancers, particularly in enhancing cellular proliferation, invasion, and metastatic capacity. Preclinical studies demonstrate that inhibition of this pathway effectively suppresses tumor initiation, progression, and metastasis. For example, targeting Notch1-Hes1 signaling inhibits proliferation in cervical cancer (91), ovarian cancer (92), and glioma (93); induces apoptosis in cervical carcinoma (70), attenuates migration/invasion in non-small cell lung cancer (NSCLC) and colon cancer models (94, 95); and enhances radiosensitivity in colorectal cancer (84). Emerging evidence highlights the critical involvement of Notch-Hes1 in immune microenvironment modulation. Mechanistically, Notch signaling upregulates Hes1 to orchestrate T-cell development and influences T-cell responses in breast cancer, suggesting its potential as an immunotherapeutic target (96). Furthermore, this pathway regulates dendritic cell-mediated anti-tumor T-cell responses and suppresses in murine models (97). The multifaceted mechanisms of Notch-Hes1 in malignancies involve EMT, angiogenesis, CSC maintenance, and dynamic tumor-stroma interactions. Continued investigation into this signaling axis will facilitate its translation as a promising therapeutic target for improving cancer prognosis.

#### 3.3.2 JAK/STAT signaling pathway

The JAK/STAT signaling pathway is an evolutionarily conserved transmembrane signal transduction mechanism critical for extracellular communication. Aberrant JAK-STAT activation and its associated genetic mutations are strongly implicated in immune dysregulation and oncogenesis (98). Mechanistically, Hes1 modulates JAK-STAT signaling by targeting STAT3, a central effector molecule. Direct binding of Hes1 to STAT3 facilitates JAK2/STAT3 complex assembly and enhances STAT3 phosphorylation (26). In rectal adenocarcinoma, an ETV4/Hes1/STAT3 signaling axis has been identified: the transcription factor ETV4 activates Hes1 transcription by binding to its promoter, which subsequently drives STAT3 phosphorylation to promote tumor proliferation and metastasis (55). Hes1 overexpression elevates STAT3 phosphorylation levels, upregulates MMP14 expression via the Hes1-STAT3-MMP14 cascade, and potentiates invasiveness in colorectal cancer (99). In HER2-overexpressing SKBR3 breast cancer cells with trastuzumab resistance, STAT3/HIF-1α axis-mediated Hes1 induction downregulates PTEN, positioning Hes1 as a pivotal node linking STAT3 signaling to PTEN suppression (100). Furthermore, cytokine/growth factor networks involving Hes1 and JAK-STAT pathways collaboratively mediate tumor immune evasion within the microenvironment (101, 102). Elucidating these interactive mechanisms will advance precision oncology by uncovering novel therapeutic targets.

#### 3.3.3 PI3K/AKT/mTOR signaling pathway

The PI3K/AKT/mTOR signaling pathway plays a central role in oncogenesis by regulating autophagy, proliferation, migration, and angiogenesis (103–105). Its activation begins with PI3K-mediated conversion of PIP2 to PIP3, which recruits AKT to the membrane for phosphorylation and subsequent activation of mTOR. Emerging evidence highlights Hes1 as a multifunctional modulator of this pathway across cancers. In gliomas, tigecycline suppresses Hes1 via miRNA-199b-5p, inhibiting PI3K/AKT signaling while elevating p21 to induce cell cycle arrest (106). Concurrently, γ-secretase inhibitor MK-0752 downregulates Notch1/Hes1 signaling, reducing AKT/mTOR phosphorylation and CXCR4 expression, thereby attenuating glioma stem cell aggressiveness (107). Mechanistically, Hes1 directly binds the PTEN promoter to repress this key AKT inhibitor (108). This regulatory axis manifests in breast cancer through Hes1-mediated PTEN suppression and AKT hyperactivation, driving tumor survival and invasion (83). In lung adenocarcinoma, GALNT2 exerts oncogenic effects via the Hes1-PTEN-PI3K/AKT cascade to amplify malignant phenotypes (109). Complementary studies demonstrate that Notch/Hes1 inhibition reduces mTORC1 signaling in gastric cancer (110), while targeting the Hes1/PTEN/AKT/mTOR axis impairs hepatocellular carcinoma progression (111). These findings collectively establish the Hes1-PI3K/AKT/mTOR crosstalk as a therapeutic nexus. Although dual-targeting strategies show promise, mechanistic variations in Hes1’s pathway regulation across cancer types demand further exploration.

#### 3.3.4 Wnt/β-catenin signaling pathway

The Wnt/β-catenin signaling pathway is often highly activated in cancer and is a key pathway regulating cell proliferation, differentiation, and tumorigenesis (112). Hes1 can regulate key genes of the Wnt/β-catenin pathway, such as β-catenin, and forms complex feedback loops with this pathway. Numerous cancer studies have revealed the close interaction between the two. For example, in colorectal cancer, inhibiting key genes of the Hes1 and Wnt pathways (such as CTNNB1, CCND1) can affect tumor cell proliferation and apoptosis (113). Additionally, RIP140, a transcriptional co-regulator of the Wnt pathway, has been found to regulate colorectal cancer cell proliferation and tumorigenesis through interaction with Hes1 (114). In ovarian cancer IGROV1 cells, both Wnt/β-catenin and Notch signaling promote cancer cell survival. Notch inhibition via DAPT reduces β-catenin levels, whereas Wnt inhibition with ICG-001 increases Hes1 expression, indicating the existence of a compensatory balance mechanism that ensures cancer cell survival (115). In glioblastoma, miR-139 inhibits Notch1/Hes1, thereby suppressing the activation of the Wnt/β-catenin pathway, thus inhibiting glioma stem cell stemness and tumorigenesis (116). Endometrial cancer studies reveal crosstalk between Notch/Hes1 and Wnt/β-catenin in differentiation: GSK-3β inhibition simultaneously upregulates Hes1 and β-catenin, driving cancer cell proliferation/migration (117). In TNBC MDA-MB-231 cells, significant Wnt/β-catenin and Notch/Hes1 crosstalk exists, making dual pathway targeting a potential therapeutic strategy (118). Therefore, future research needs to delve into the specific regulatory mechanisms of Hes1 and the Wnt/β-catenin pathway in various tumors, as well as their potential in clinical therapy.

#### 3.3.5 Hh/Gli signaling pathway

The Hedgehog/Gli (Hh/Gli) signaling pathway plays a crucial role in embryonic development and tissue homeostasis. It also creates favorable conditions for tumor progression and metastasis by regulating tumor cell growth, differentiation, and the immune microenvironment (119, 120). Hes1, as a key target gene of the Notch signaling pathway, exhibits significant cross-regulation with the Hh/Gli pathway. Together, they influence tumor cell proliferation, differentiation, and drug resistance, driving tumor progression and treatment resistance (121). Specific mechanisms have been revealed in different tumors: in pancreatic β cells, excessive activation of Hh signaling upregulates precursor markers such as Hes1 and SOX9, induces β cell dedifferentiation, and ultimately promotes the formation of undifferentiated pancreatic tumors (122); in multiple myeloma, the Gli inhibitor GANT61 inhibits the expression of Notch1, Jagged1/2, and Hes1 in a dose- and time-dependent manner, suppressing proliferation and promoting apoptosis by blocking Notch signaling (45); in glioblastoma (GBM), Hes1 can directly bind to the first intron of the Gli1 gene to inhibit its transcription, while inhibition of Notch signaling leads to compensatory activation of the Hh pathway (which is associated with the loss of Hes1 binding at the Gli1 site), enabling tumor cells to maintain survival and proliferation capabilities by upregulating Hh signaling during Notch inhibition, and simultaneous targeting of Notch and Hh pathways more significantly induces apoptosis, reduces cell growth, and inhibits colony formation ability compared to single therapies (123). In summary, Hes1 may serve as a key intersection point between the Notch and Hh/Gli signaling pathways. Future research should delve deeper into its potential as a therapeutic target, particularly in the context of combined targeting strategies.

### 3.4 Epigenetic mechanisms of Hes1 in tumors

The expression and function of Hes1 are regulated by various epigenetic mechanisms, including DNA methylation modifications, histone modifications, and non-coding RNA regulation, which profoundly influence its role in tumorigenesis, progression, and treatment. DNA methylation is one of the important epigenetic regulatory mechanisms of Hes1, and can regulate its expression directly or indirectly. In hepatocellular carcinoma, hypomethylation of the promoter region of KK-LC-1 (a cancer/testis antigen) leads to its upregulation, which in turn promotes tumor progression by activating the Notch1/Hes1 signaling pathway. Blocking Notch signaling with the γ-secretase inhibitor DAPT can attenuate the malignant phenotypes induced by KK-LC-1 overexpression (124). Additionally, the promoter region of Hes1 itself is directly regulated by methylation. In colorectal cancer (CRC), hypomethylation of the Hes1 promoter region results in its significant upregulation, and its high expression status is closely associated with tumor malignancy, lymph node metastasis, and advanced clinical stages, indicating a worse prognosis for CRC patients with Hes1 hypomethylation (125).

Histone modification is another critical regulatory mechanism involving various histone-modifying enzymes. In colorectal cancer, the STRAP protein competitively inhibits the assembly of the histone methyltransferase PRC2 complex (containing EZH2 and SUZ12), reducing the enrichment of H3K27me3 at the promoter regions of NOTCH pathway-related genes, including Hes1, thereby activating Hes1 expression and promoting CSC self-renewal and tumorigenesis (126). In breast cancer, imatinib inhibits the acetyltransferase activity of p300, leading to decreased levels of H3K18Ac and H3K27Ac, thereby downregulating Hes1 expression and inhibiting EMT (127).

Non-coding RNAs primarily regulate Hes1 through post-transcriptional mechanisms. In medulloblastoma, miR-199b-5p directly targets Hes1 mRNA to inhibit its expression, thereby suppressing the proliferation of tumor stem cells (128, 129). Conversely, the methylation of miRNA-9 leads to its downregulation, promoting Hes1 expression and subsequently enhancing tumor cell proliferation and differentiation (130). In hepatocellular carcinoma, overexpression of miR-760 downregulates the expression of Notch1 and Hes1, increasing the sensitivity of tumor cells to the chemotherapeutic drug doxorubicin (131). Additionally, epigenetic mechanisms interact with each other or with other signaling pathways, forming a complex regulatory network. In uveal melanoma, lncRNA PAUPAR downregulates Hes1 expression by inhibiting histone H3K4 methylation, thereby suppressing tumorigenesis and metastasis (132); in glioblastoma, there is a feedback regulation between Smarcd1, a component of the chromatin remodeling complex SWI/SNF, and the Notch1/Hes1 axis, where Smarcd1 overexpression reduces Notch1 expression, and Notch1 knockdown conversely increases Smarcd1 expression by inhibiting Hes1, ultimately inhibiting the malignant phenotype of the tumor (133).

Given the critical role of Hes1 in tumors and its complex epigenetic regulatory network, targeted intervention strategies (such as DNA methylation inhibitors, histone modification regulators, and microRNA therapies) demonstrate promising clinical applications. For instance, in medulloblastoma, the use of DNA methylation inhibitors (e.g., 5-aza-deoxycytidine) to restore miR-199b-5p expression can inhibit tumor stem cells by negatively regulating Hes1 (128). In colorectal cancer, silencing STRAP can form inhibitory chromatin domains to suppress the activation of the Notch1-Hes1 axis, weaken CSC self-renewal, and enhance chemosensitivity (126). Future research should delve into the specific epigenetic regulatory mechanisms of Hes1 and explore its clinical translation potential, aiming to provide more effective solutions for tumor treatment.

### 3.5 Immune functions of Hes1 within the TME

**Figure 4:**
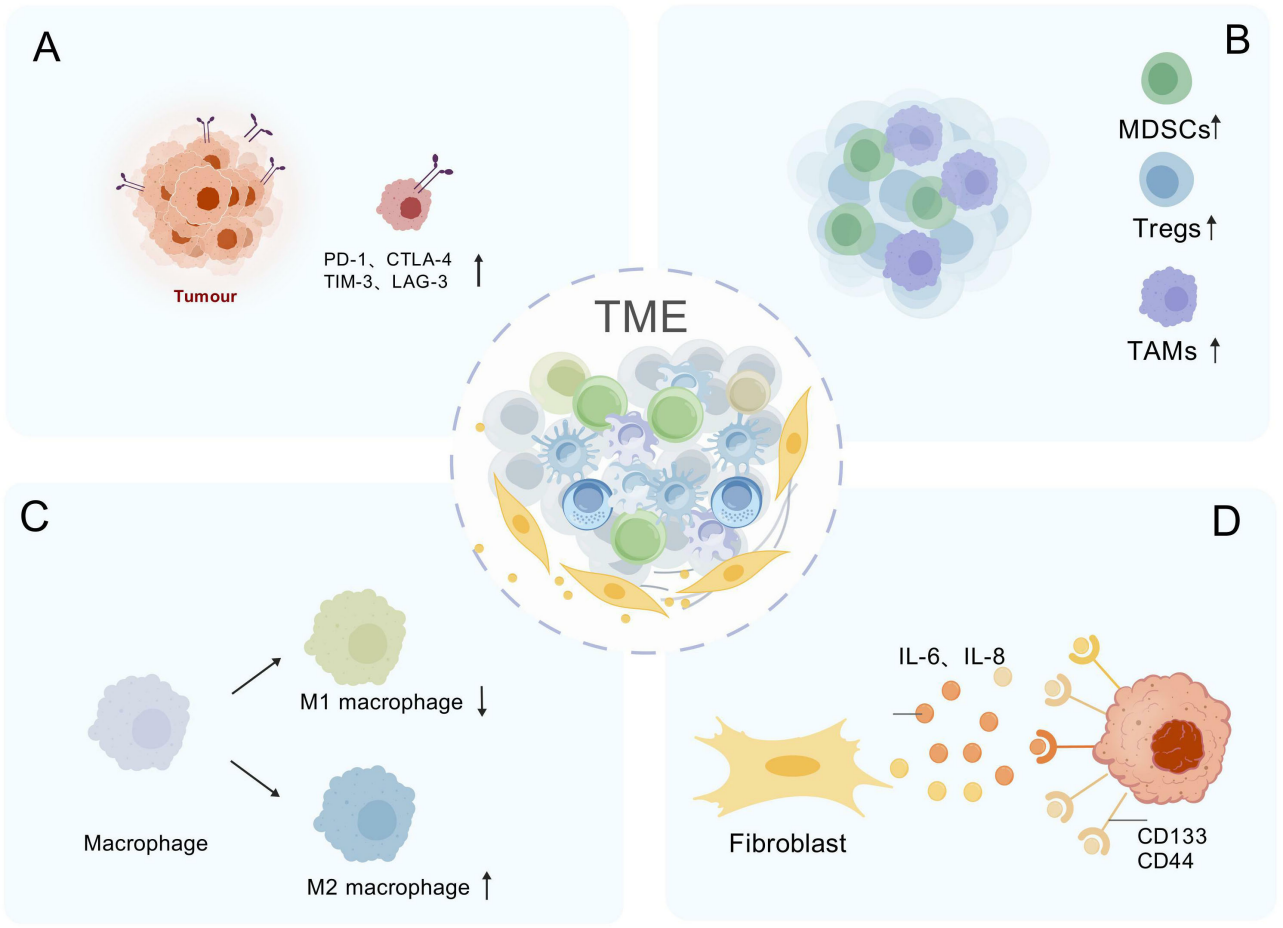
Hes1-Mediated Immunosuppressive Network in the Tumor Microenvironment. **(A)** Immune checkpoint induction: Hes1 upregulates PD-1, CTLA-4, TIM-3, and LAG-3 to suppress T-cell activity. **(B)** Immunosuppressive cell expansion: Hes1 recruits immunosuppressive cells (MDSCs/Tregs) to amplify inhibition. **(C)** Induction of macrophage polarization: Hes1 polarizes TAMs toward M2 phenotype. **(D)** Cancer stem cell (CSC) activation: Hes1 activated via IL-6/IL-8 signaling drives CD133^+^/CD44^+^ cancer stem cell expansion and induces immune suppression. "Created with BioGDP.com" (Note: This platform was used for data visualization and is acknowledged in the Acknowledgments section.)

Hes1 plays a crucial role in tumor immune escape by influencing immunosuppressive cells and immune checkpoint molecules within the TME (**Figure 4**). The high expression of Hes1 is closely associated with the enrichment of various immunosuppressive cells, aiding tumors in evading immune system surveillance. Tumor-associated factors (TAFs) upregulate Hes1 in tumor-associated macrophages (TAMs), inducing the expression of arginase-1 (Arg1), which depletes arginine, thereby suppressing T cell activation and function, and promoting immune escape (88); Hes1 interferes with lactate metabolism by inhibiting the transcription of the lactate transporter MCT2, enhancing TAM maturation and immunosuppressive function (134); High expression of Hes1 is associated with M2-type TAM polarization (135), and zinc finger protein 746 (ZNF746) upregulates Hes1 by activating the Jagged1/Notch pathway, driving M2-type macrophage polarization to promote breast cancer progression (136). Conditional knockout of Hes1 in TAMs enhances the infiltration and activation of cytotoxic T cells, significantly inhibiting tumor growth (88). Hes1 also drives the expansion of other immunosuppressive cells. For instance, in head and neck squamous cell carcinoma (HNSCC), its expression is positively correlated with the enrichment of myeloid-derived suppressor cells (MDSCs) and regulatory T cells (Tregs). The Notch-Hes1 pathway promotes the generation of MDSCs/Tregs, thereby suppressing anti-tumor immune responses (137). However, the role of Hes1 exhibits tissue specificity. For instance, in KRAS-mutated colorectal cancer, the loss of Hes1, while promoting the remodeling of the ECM, enhances the polarization of M2-type macrophages and the expression of immunosuppressive factors such as IL-10 (138). This indicates that Hes1 also influences the immunosuppressive state of the TME by regulating the composition and function of the ECM.

The occurrence and progression of tumors are closely associated with immune evasion, in which immune checkpoints play a significant role (139). The co-expression of Hes1 and ARID3B is associated with poor prognosis in colorectal cancer, and they may synergistically recruit histone-modifying enzymes (such as KDM4C) to regulate chromatin structure, thereby activating PD-L1 immune checkpoint transcription and inhibiting T cell activity (140). Hes1 is also associated with the expression of other checkpoint molecules such as CTLA4, TIM3, and LAG3. Inhibition of the Notch/Hes1 signaling pathway using γ-secretase inhibitors significantly reduces the expression of these checkpoints in head and neck squamous cell carcinoma (137). Additionally, Hes1 can influence the TME through the regulation of CSCs and is closely related to immune evasion. Colorectal cancer-associated myofibroblasts activate Hes1 through the secretion of IL-6 and IL-8, promoting the expansion of CD133 (+) and CD44 (+) CSCs and suppressing immune cell activity (79); in a hypoxic microenvironment, HIF-1α activates the Notch1/Hes1 pathway, inducing the transformation of prostate cancer cells into a stem cell phenotype (141).

In summary, Hes1 plays a central role in the formation of an immunosuppressive TME and tumor immune escape by regulating TAMs, MDSCs, Tregs, immune checkpoints, ECM remodeling, and cancer stem cells, making it a highly promising therapeutic target. Furthermore, Hes1 can serve as an immune microenvironment-related prognostic marker: in prostate cancer, its high expression is associated with M2-type TAMs and enhances tumor proliferation and invasion (142); in TNBC, Hes1 expression levels are significantly correlated with immune cell infiltration and immunosuppressive status (143). In the future, it is necessary to delve into the regulatory network of Hes1 in the TME and develop targeted strategies to improve tumor immunotherapy efficacy.

## 4 The tumor type-specific role and clinical significance of Hes1

The expression patterns, clinicopathological characteristics, and underlying mechanisms of Hes1 in various human cancers are summarized in **Tables 1**, **2**.

**Table 1:** Hes1 expression patterns, clinicopathological characteristics, and prognostic significance across malignant tumors.

Disease type	Number of cases	Expression	Clinicopathological characteristics	Prognosis	Refs.
Colorectal cancer (CRC)	320	high	increased incidence of distal metastasis; worse OS	poor	(81)
Colorectal cancer (CRC)	74	high	shorter survival in the subset of patients with stage I-III CRC	poor	(99)
Colorectal cancer (CRC)	2775	loss of nuclear	female sex, right-sided location, BRAFV600E mutation, microsatellite instability, larger tumor size, and significantly worse survival	poor	(150)
Gastric cancer (GC)	269	high	overall survival	poor	(77)
Pancreatic cancer (PC)	72	high	shorter OS and DFS; is essential for chemoresistance induced by stellate cells	poor	(199)
Pancreatic cancer (PC)	31	high	overall survival	poor	(200)
Breast cancer (BC)	150	high	advanced TNM stage, positive node metastasis, negative estrogen receptor expression and triple-negative status.	poor	(83)
Squamous cervical carcinoma (SCC)	109	high	tumor size and invasive depth in early-stage SCC patients undergoing surgery	poor	(201)
Ovarian cancer (OC)	61	high	shorter DFS and OS	poor	(202)
Lung adenocarcinoma	89	high	worse five-year OS	poor	(203)
Lung cancer	88	high	SCLC more strongly expresses the neuroendocrine phenotype; LCNEC shows characteristics more similar to the bronchial epithelium phenotype	/	(204)
Small cell lung cancer (SCLC)	46	high	/	/	(205)
Hepatocellular carcinoma (HCC)	108	low	Shorter median OS and recurrence time	poor	(206)
Chronic lymphocytic leukemia (CLL)	60	high	Clinicopathological Staging;LDH concentration	poor	(65)
Acute myeloid leukemia (AML)	30	low	/	/	(182)

OS, overall survival; DFS, disease-free survival; SCLC, small cell lung cancer; LCNEC, large cell neuroendocrine carcinoma; HCC, hepatocellular carcinoma; CLL, chronic lymphocytic leukemia; AML, acute myeloid leukemia; LDH, lactate dehydrogenase **Table 2**.

**Table 2:** Molecular mechanisms and functional roles of Hes1 in malignant tumors.

Disease type	Expression	Mechanism	Function	Refs.
Colorectal cancer (CRC)	Upregulated	Hes1-STAT3-MMP14	increases cell invasion ability	(99)
Colon adenocarcinoma (COAD)	Upregulated	ETV4-Hes1-STAT3	enhances proliferation and metastasis	(55)
Colorectal cancer (CRC)	Upregulated	Hes1-EMT	enhances invasiveness and metastasis	(81)
Breast cancer (BC)	Upregulated	Hes1-PTEN-AKT; Hes1-EMT	promotes proliferation and invasion	(83)
Triple-negative breast cancer (TNBC)	Upregulated	Hes1-Slug; Hes1-Slug-EMT	promotes BCSC stemness properties and invasion	(28)
Cervical Carcinoma (CC)	Upregulated	Hes1/Hes5-Hash1	inhibits differentiation; promotes cell proliferation	(69)
Endometrial cancer (EC)	Upregulated	FGFR-Hes1; FGFR-AKT-Hes1	activates cell proliferation	(46)
Non-small cell lung carcinoma (NSCLC)	Upregulated	NICD- Hes1	promotes cell proliferation	(207)
Non-small cell lung carcinoma (NSCLC)	Upregulated	ST6Gal-I-Notch1-Hes1-MMPs	mediates invasiveness and tumorigenicity	(95)
Lung adenocarcinoma (LUAD)	Upregulated	GALNT2-Notch/Hes1-PTEN-PI3K/Akt	increases proliferation and metastasis	(109)
Chronic lymphocytic leukemia (CLL)	Upregulated	Hes1-Notch1; Notch1-Hes1-PTEN	promotes proliferation; represses apoptosis; increases S-phase arrest	(65)
T-cell acute lymphoblastic leukemia (T-ALL)	Upregulated	Notch1-Hes1-BBC3	increases cell survival	(68)
Acute myeloid leukemia (AML)	Upregulated	Hes1-P21	inhibited cell growth; induces apoptosis;suppresses AML formation *in vivo*	(182)
Chronic myelogenous leukemia (CML)	Upregulated	Hes1-NF-κB-MMP-9	enhances proliferation of leukemic cells	(180)

EMT, epithelial-mesenchymal transition; BCSC, breast cancer stem cell; TNBC, triple-negative breast cancer; COAD, colon adenocarcinoma; NSCLC, non-small cell lung cancer; LUAD, lung adenocarcinoma; T-ALL, T-cell acute lymphoblastic leukemia; CML, chronic myelogenous leukemia.

### 4.1 Colorectal cancer

Colorectal cancer (CRC) is one of the most prevalent malignancies, with a subset of cases having hereditary risk (144, 145). Hes1 is extensively expressed in the basal region of normal intestinal crypts and CRC tissues (73, 146), yet its role in CRC progression presents contradictions. Numerous studies have reported elevated Hes1 expression in CRC: mRNA levels in CRC tissues are generally higher than those in adjacent normal tissues (38, 73, 125, 147); immunohistochemistry has demonstrated a gradient increase in average Hes1 immunoreactivity from normal mucosa to adenoma to cancerous regions (79); epigenetic analysis indicates that low methylation of the Hes1 promoter correlates with its overexpression and is significantly associated with histological grade progression, lymph node metastasis, and poor prognosis (125). However, some studies have reached opposing conclusions: Hes1 expression is often absent in right-sided CRC and precancerous lesions (sessile serrated adenoma/polyps) (146, 148); a large-scale cohort study (n=327) found that low Hes1 expression is significantly linked to increased tumor size, lymphovascular invasion, and distant metastasis, with patients exhibiting low HES-1 expression showing significantly lower overall survival rates compared to those with high HES-1 expression (149); immunohistochemistry analysis of 2775 CRC cases revealed that the absence of Hes1 nuclear expression (17.0%) is significantly associated with microsatellite instability, BRAFV600E mutation, and reduced 5-year survival rates (150).

At the cellular model level, Hes1 demonstrates cancer-promoting characteristics: overexpression of Hes1 enhances the invasive ability of CaCo2/SW48 cells, while knockout induces β-galactosidase-mediated cellular senescence (99); it activates aerobic glycolysis via the IGF2BP2-GLUT1 axis, facilitating energy metabolism reprogramming in CRC (147); it preserves cancer stem cell properties and activates the ABC transporter family, mediating 5-FU resistance (73, 82, 151). Animal experiments further corroborate the tumorigenicity (73) and metastatic potential (81)of tumors with high Hes1 expression. However, specific microenvironments can alter its function. For example, RIP140 overexpression transforms Hes1 from a proliferative to an antiproliferative phenotype by suppressing its mitotic activity. Notably, in patients with high RIP140 expression, elevated Hes1 levels correlate with improved survival (114). The existing discrepancies regarding Hes1 may stem from spatial heterogeneity, molecular interaction networks, and subtype specificity. Variations in Hes1 nuclear/cytoplasmic localization influence its function, and the absence of nuclear expression may induce an immunosuppressive microenvironment by activating the IL6/IL10 pathway (138); dynamic regulation of Hes1 function is mediated by factors such as autonomous oscillatory expression characteristics (21) and the involvement of regulatory factors like RIP140; inherent differences in Notch signaling activity and microsatellite status may exist between right-sided and left-sided CRC (138, 150). These factors indicate that Hes1 is more than just an oncogene or tumor suppressor. Instead, it acts as a “molecular rheostat” in CRC, with its ultimate effect highly dependent on the pathological context.

### 4.2 Breast cancer

Breast cancer (BC) is the most common malignancy among women, characterized by high heterogeneity, and is one of the leading causes of cancer incidence globally (152, 153). Immunohistochemical analysis of 150 primary breast cancer specimens revealed weak nuclear expression of Hes1 in 5 cases of ductal carcinoma *in situ* (DCIS), while its expression was significantly upregulated in 53 cases of invasive ductal carcinoma (IDC) (35.3%) (83). The overexpression of Hes1 is significantly associated with advanced TNM staging, lymph node metastasis, and estrogen receptor (ER)-negative status, suggesting its critical role in breast cancer progression and metastasis.

In TNBC, Hes1 expression is significantly higher than in other subtypes and is closely related to poor prognosis in TNBC patients (28, 83). Mechanistically, Hes1 maintains the stemness of breast cancer stem cells (BCSCs) by directly activating Slug transcription, thereby enhancing the aggressiveness and treatment resistance of TNBC (28). Notably, Hes1 expression is significantly upregulated in tamoxifen-resistant patients compared to tamoxifen-sensitive cases, and its overexpression is also associated with N staging, nipple involvement, and poorer prognosis in patients with DSF, indicating that Hes1 plays a vital role in the progression and drug resistance of ER-positive breast cancer (87). Hes1 regulates breast cancer cell proliferation, EMT, and invasion through multiple pathways such as Notch, AKT, STAT, and HIF-1α (28, 59, 83, 87, 100). The Notch pathway promotes breast cancer proliferation and invasion by upregulating Hes1 (154, 155), while the STAT3/HIF-1α/Hes1 axis mediates trastuzumab resistance in HER2-positive breast cancer by inhibiting PTEN (100). In the TME, extracellular vesicles (EVs) carrying miR-887-3p enhance chemoresistance by activating Notch1/Hes1 signaling (156). Under hypoxic conditions, both mRNA and protein levels of Hes1 are significantly upregulated, contributing to angiogenesis, metastasis, and treatment resistance. *In vitro* experiments have confirmed that knocking down Hes1 can inhibit hypoxia-induced breast cancer cell proliferation and invasion (157). Therefore, Hes1 is a core regulatory factor in the development and treatment resistance of breast cancer, and targeting Hes1 and its related pathways may provide new strategies for precision treatment of breast cancer.

### 4.3 Gastric cancer

Gastric cancer (GC) ranks as the fifth most common malignancy globally and is the third leading cause of cancer-related deaths (158). Immunohistochemistry and immunofluorescence analysis of 269 gastric cancer tissue samples revealed that 89% of them exhibited positive Hes1 expression (moderate to strong staining) (77). Clinical studies have indicated that Hes1 positivity is associated with reduced overall survival rates among patients. Consistent results from both *in vitro* and *in vivo* experiments have demonstrated significant upregulation of Hes1 expression in gastric cancer cell lines and tissues (74, 77, 110). Downregulation of Hes1 through DAPT or siRNA can inhibit Snail expression, impair EMT (74, 159), and attenuate the proliferation, migration, invasion, and chemoresistance of gastric cancer cells. These findings suggest that high Hes1 expression drives the malignant progression of gastric cancer and may serve as a biomarker for poor prognosis.

Mechanistically, the isoforms of the HLH transcription factor Id1 (Id1a and Id1b) bind to Hes1 in a dose-dependent manner, blocking its association with the N-box. Dysregulation of the Id1-Hes1 feedback loop may contribute to the differentiation arrest and abnormal proliferation of gastric cancer cells (160). Furthermore, Hes1 collaborates with the Wnt/β-catenin, Hedgehog, and mTOR pathways to promote gastric cancer progression, indicating potential for combination therapy. For instance, γ-secretase inhibitors (GSIs) can simultaneously suppress Notch/Hes1 and Wnt/β-catenin signaling, effectively inhibiting the proliferation, migration, and invasion of CD44(+) gastric cancer stem cells (GCSCs) and inducing apoptosis (77). Combined inhibition of Hes1 and Smo reverses the tumor sphere formation and increase in invasive cell populations caused by miR-7-5p suppression, highlighting the synergistic role of Notch and Hedgehog pathways in GCSC invasion (75). Co-activation of Notch and mTOR pathways drives gastric cancer proliferation. Notably, inhibiting Notch reduces mTORC1 signaling activity, supporting a combined targeted therapy strategy (110). Preclinical studies have shown that blocking Notch1-Hes1 signaling can reverse chemoresistance in gastric cancer (74, 161). Knockdown of Hes1 significantly impairs GCSC stemness and enhances sensitivity to EGFR-TKIs (74), suggesting that combining Notch inhibitors with EGFR/HER2-targeted drugs may improve therapeutic efficacy. Hes1 emerges as a critical factor regulating gastric cancer stem cell properties, malignant behaviors, treatment resistance, and prognosis. Future research should delve deeper into its molecular mechanisms and develop precision therapy strategies targeting Hes1-related pathways

### 4.4 Pancreatic cancer

Pancreatic cancer, one of the most aggressive tumor types, involves multistage biological processes in its initiation and progression. This complexity underscores the importance of exploring novel biomarkers and molecular therapeutic targets in current research (162, 163).Hes1 expression in the early embryonic pancreas is continuous (164) and plays a crucial role in pancreatic formation (35, 165). In the adult pancreas, Hes1 expression is primarily detected in terminal duct cells or centroacinar cells (164), and the absence of Hes1 does not lead to phenotypic changes (166). Notably, although Hes1 is not expressed in normal acinar cells, it becomes activated during the oncogenic transformation process, specifically during acinar-to-ductal metaplasia (ADM), and remains expressed through pancreatic intraepithelial neoplasia (PanIN) until the development of pancreatic ductal adenocarcinoma (PDAC). PanIN serves as a precursor to invasive pancreatic cancer (167). Mechanistic studies have revealed that Hes1 drives the transition from ADM to PanIN by regulating the gene network associated with acinar-to-ductal reprogramming (ADR) (168). *In vivo* studies further confirm that the absence of Hes1 significantly inhibits KRASG12D-driven PanIN formation in mice, maintaining PanIN in a low-grade state and effectively blocking its progression to PDAC. However, it’s important to note that Hes1’s regulatory role is stage-dependent: knocking out Hes1 before PanIN formation can inhibit lesion development, while knocking it out after PanIN formation paradoxically promotes lesion expansion (166). Additionally, the absence of Hes1 can promote PDAC progression by upregulating the expression of Muc5ac, a member of the mucin family, and enhancing EMT (166). These studies suggest that Hes1 plays a dynamically regulatory role in the evolution of pancreatic cancer, and its shift between tumor-promoting and tumor-suppressing functions depends on the stage of tumorigenesis and the differential regulation of downstream target genes. This spatio-temporal specificity of regulation indicates that intervention strategies targeting Hes1 require precise control of the therapeutic time window. Future research should further elucidate: 1) the signal regulation network of Hes1 in different tumor microenvironment; 2) the molecular switching mechanism underlying its stage-dependent role; and 3) the synergistic mode of action with driver genes such as KRAS. The resolution of these key issues will provide a theoretical basis for precision treatment of pancreatic cancer.

### 4.5 Lung cancer

As the leading cause of cancer-related deaths worldwide (169), lung cancer’s occurrence and development are closely linked to smoking. Studies have indicated that in cigarette smoke exposure models, the expression of Notch1 and Hes1 proteins is significantly upregulated in the nuclei of lung adenocarcinoma A549 cells, suggesting that tobacco carcinogens may promote lung adenocarcinoma development by activating the Notch1-Hes1 signaling axis (170). Further mechanistic studies have revealed that the Notch/Hes1 signaling network participates in the malignant progression of lung cancer through multiple molecular pathways: the Notch1/Hes1/p-STAT3 signaling axis regulates the self-renewal capacity of lung CSCs (171), while the Notch1/Hes1/matrix metalloproteinases (MMPs) cascade mediates the invasive and metastatic properties of NSCLC (95).

At the clinical translation level, Hes1 has been identified as a stem cell marker for EGFR-mutant positive NSCLC (172), with its expression level closely correlating with treatment response. Abnormal activation of the Notch pathway is associated with acquired resistance to EGFR-TKI. Preclinical studies have demonstrated that combining gamma-secretase inhibitors (GSI) with EGFR-TKI can overcome drug resistance, offering a new strategy for targeted therapy (173). Focusing on the high-proportion subtype of lung adenocarcinoma (LUAD), research has found that Hes1 expression is significantly elevated in EGFR-TKI-resistant cells. Introducing the Notch4^ΔL12_16^ mutation can reverse drug resistance, possibly by inducing a reduction in the intracellular domain (NICD4), weakening its competitive binding with p-STAT3 to the Hes1 promoter, enhancing p-STAT3’s transcriptional repression of Hes1, and ultimately increasing tumor sensitivity to EGFR-TKI (85). Breakthroughs have also been made in the field of small cell lung cancer (SCLC) research, where Hes1 has been identified as a key mediator of chemotherapy resistance. MYCN activates Hes1 transcription by directly binding to its promoter and synergistically forms a positive feedback loop with Notch signaling. Targeted inhibition of Hes1 can effectively reverse the drug-resistant phenotype induced by MYCN overexpression (174). These discoveries untangle the central role of Hes1 in lung cancer drug resistance: by maintaining CSC stemness, regulating epigenetic remodeling, and forming interactive dialogues with other oncogenic pathways (such as MYCN and STAT3), Hes1 contributes to the construction of multi-layered drug resistance barriers. Hes1 exhibits networked regulatory characteristics in lung cancer progression, and its potential as a therapeutic target urgently needs to be further explored. Future research should focus on addressing: 1) the association between the spatiotemporal dynamic expression of Hes1 and tumor heterogeneity; 2) the molecular basis of its synergistic effects with driver mutations such as KRAS/EGFR; 3) the development of combination therapy strategies based on the Hes1 signaling node.

### 4.6 Leukemia

Leukemia comprises multiple subtypes involving numerous critical molecular processes, rendering it a highly aggressive and challenging disease (175). Hes1 is overexpressed in B lymphocytes from peripheral blood samples of patients with chronic lymphocytic leukemia (CLL), and its expression positively correlates with the clinicopathological staging of CLL patients (65). The development of CLL has been demonstrated to be triggered by enhanced PI3K activity resulting from the downregulation of PTEN (176). Overexpression of Hes1 can bind to the promoter region of PTEN and decrease its expression (65). *In vivo* studies have confirmed that the IDH1-R132H mutation upregulates Hes1 expression and downregulates PTEN expression by activating the Notch1 pathway, thereby activating the PI3K/AKT pathway and promoting malignant behavior in T-cell acute lymphoblastic leukemia (T-ALL) cells (177). In the microenvironment formed by CLL cells and HS-5 stromal cells, transcriptional activation of Hes1 leads to demethylation of H3K27me3, protecting CLL cells from apoptosis and enhancing their survival (178). Hes1 is often overexpressed during the blast crisis phase of chronic myeloid leukemia (CML) (179). The mechanism may involve the activation of NF-κB, which upregulates MMP-9 and promotes the development of CML during the blast crisis phase (180). Under normal circumstances, Hes1 is lowly expressed in acute myeloid leukemia (AML) (179), and the Notch pathway exists in AML but is not activated (181). However, activated and overexpressed Hes1 can play an unexpected anti-tumor role in AML. Retroviral-induced activation of Hes1 *in vitro* can lead to growth arrest and apoptosis of AML cells, while overexpression of Hes1 *in vivo* can inhibit the formation of AML (182). Furthermore, Hes1 can directly bind to the promoter of the FLT3 gene and downregulate its activity, inhibiting the progression of AML (183). Overexpressed Hes1 can inhibit fatty acid oxidation (FAO), improving chemotherapy resistance induced by SIRT3 SUMOylation in AML (184). The expression and function of Hes1 vary across different subtypes and developmental stages of leukemia. The contradictory role of Hes1 in suppressing or promoting cancer in leukemia presents both a therapeutic challenge and a potential breakthrough. Future research should focus on achieving precise spatiotemporal intervention through multi-omics integration and dynamic functional analysis.

## 5 Therapeutic strategies targeting Hes1 in tumors

Hes1 plays a pivotal role in the genesis, progression, and therapeutic resistance of various tumors, making the targeted therapy and combination strategies against Hes1 a research hotspot. The activity of Hes1 primarily depends on its dimerization and DNA-binding capabilities. Studies have shown that natural products such as gallic acid can effectively inhibit Hes1 dimerization, thereby suppressing its transcriptional repression function (185). The synthetic DNA-binding inhibitor PIP-RBPJ-1 can specifically bind to the Hes1 promoter region, inhibiting its transcriptional activity (186). Additionally, the protein level of Hes1 is regulated by the ubiquitin-proteasome system. Research has found that RASSF1A promotes the ubiquitination and degradation of Hes1 by recruiting the SUMO-targeted E3 ligase SNURF/RNF4 (187), providing a theoretical basis for the development of drugs based on Hes1 degradation. Small molecule inhibitors are one of the effective strategies for directly targeting Hes1. For instance, in preclinical studies of fusion-negative rhabdomyosarcoma (FN-RMS), the small molecule inhibitor JI130 significantly inhibited tumor cell growth and promoted apoptosis by suppressing Hes1 expression (188). Another small molecule, perhexiline (a mitochondrial carnitine palmitoyltransferase-1 inhibitor), has also been found to possess Hes1 antagonist activity, demonstrating significant anti-tumor effects in leukemia models (68). Gene knockout and RNA interference techniques are commonly used methods for studying Hes1 function and validating its targeting value. In intrahepatic cholangiocarcinoma (ICC), Hes1 gene knockout significantly inhibits tumorigenesis and progression (189). Conditional knockout of Hes1 in mouse models markedly slows tumor growth and increases the infiltration and activation of cytotoxic T lymphocytes (CTLs) in the TME (88), suggesting that targeting Hes1 may be an effective immunotherapy strategy. The use of epigenetic regulators is also one of the strategies for targeting Hes1 therapy. For instance, miR-199b-5p inhibits the proliferation and metastasis of tumor stem cells in medulloblastoma by negatively regulating Hes1; demethylating agents (such as 5-aza-deoxycytidine) can restore the expression of miR-199b-5p, thereby suppressing Hes1 and reducing the population of tumor stem cells (128). In TNBC MDA-MB-231 cells, forced expression of miR-181c-5p can negatively regulate the Notch1 oncogenic signaling by binding to the 3’ UTR target site of NOTCH1, suppressing Hes1 expression, and providing a novel therapeutic approach for TNBC (190).

As a key regulator of the Notch signaling pathway, Hes1 exhibits extensive crosstalk with other signaling pathways, which endows the combined targeting strategy with potential advantages. γ-Secretase inhibitors (GSIs) represent a classic strategy for targeting the Notch pathway. DAPT can downregulate the expression of Notch1 and Hes1 in a dose- and time-dependent manner, inducing growth inhibition and apoptosis in ovarian cancer A2780 cells (191). In ovarian cancer, GSI MK-0752 combined with chemotherapeutic agents (such as cisplatin) has demonstrated enhanced anti-tumor efficacy (192). In gastric cancer, simultaneous blockade of Notch (using DAPT) and PI3K/Akt signaling (using LY294002) can synergistically inhibit the expression of Notch1, Hes1, and p-Akt, significantly suppressing tumor metastasis (193). In endometrial cancer and TNBC, there exists crosstalk between the Notch/Hes1 and Wnt/β-catenin pathways, and dual targeting of these pathways represents a potential therapeutic direction (117, 118). In EGFR-mutant lung adenocarcinoma, elevated levels of Hes1 protein are associated with shorter progression-free survival in patients treated with tyrosine kinase inhibitors (TKIs); however, the combination of Notch inhibitors with TKIs (such as gefitinib and osimertinib) significantly reduces Hes1 expression and overcomes drug resistance (194). In head and neck squamous cell carcinoma (HNSCC), the CHK1/2 inhibitor Prexasertib enhances the cytotoxicity of cisplatin and radiotherapy by inhibiting the Notch signaling pathway, including Hes1 (195) Immunohistochemistry of HNSCC tissues reveals that elevated Hes1 is associated with myeloid-derived suppressor cells (MDSCs), tumor-associated macrophages (TAMs), regulatory T cells (Tregs), and immune checkpoint molecules (PD1, CTLA4, TIM3, LAG3); inhibition of the Notch signaling pathway using GSIs (such as GSI-IX, DAPT) may reduce these immunosuppressive cells and molecules (137). It is noteworthy that Hes1 knockout mice exhibited more significant suppression of tumor growth following treatment with combined immune checkpoint inhibitors, such as anti-PD-1 antibodies (88), further highlighting the potential of Hes1 as a synergistic target in immunotherapy. In summary, targeting Hes1 demonstrates significant potential in cancer therapy.

## 6 Conclusion and future perspectives

As a core member of the bHLH transcription factor family, Hes1 plays a pivotal role in regulating biological processes such as cell differentiation, cycle arrest, programmed death, and stem cell maintenance. This review systematically analyzes the structure and modification characteristics of Hes1, delves into its regulatory mechanisms, and elucidates its multifaceted functions in tissue development abnormalities and malignant tumor progression at the molecular level. The focus is on the latest research advancements in Hes1-driven tumorigenesis and development, providing a theoretical framework for anti-tumor treatment strategies targeting Hes1.

Hes1 participates in the malignant progression of tumors through a complex regulatory network, involving multiple oncogenic pathways such as inducing EMT and maintaining tumor stem cell properties, which has become a research hotspot in the field of cancer (81, 196, 197). Hes1 possesses unique structural and biological functions and engages in the pathophysiology of cancer through various molecular mechanisms and signal interactions. As mentioned in the text, abnormal high expression of Hes1 exhibits significant oncogenic properties in various solid tumors. According to clinical data analysis, the upregulation of Hes1 is positively correlated with poor prognosis in patients with malignant tumors. Conversely, the loss of Hes1 expression is also associated with invasive phenotype transformation. These findings suggest that Hes1 may serve as a potential marker for early tumor screening, prognosis prediction, and treatment monitoring. Moreover, they indicate that Hes1’s functionality is highly dependent on tissue-specific transcription factors and microenvironmental signals, as well as the compensatory activation of other survival pathways or the heterogeneity of epigenetic modifications.

Notably, Hes1 also plays a key role in shaping the tumor immune microenvironment (TME). Studies show that Hes1, through Notch signaling or other pathways, promotes the recruitment, differentiation, or functional activation of immunosuppressive cells while inhibiting the activity of effector T cells, thereby creating a microenvironment conducive to tumor immune escape (88). Furthermore, Hes1 expression levels have been found to correlate with the expression of immune checkpoint molecules, suggesting it may influence tumor cell sensitivity to immune checkpoint inhibitor therapy. Therefore, as mentioned in the targeted strategies, a deeper understanding of Hes1’s role in tumor immune regulation holds significant implications for developing novel combination immunotherapy strategies.

Hes1 participates in the self-renewal and drug resistance formation of CSCs through both the canonical Notch pathway and other non-canonical pathways. By targeting the function of Hes1 and its related signaling pathways, new therapeutic options for various cancers may emerge. Currently, most therapeutic strategies targeting Hes1 are still in the preclinical research stage. Developing treatment plans based on the expression level of Hes1 and related biomarkers in patients’ tumors has the potential to improve treatment effectiveness and tolerability. Furthermore, the crosstalk between Hes1 and other signaling pathways (JAK/STAT, PI3K/AKT/mTOR, Wnt/β-catenin, Hedgehog/Gli) further expands its functional dimensions, influencing tumor progression. This crosstalk also extends to immune-related pathways, collectively shaping the tumor’s immune phenotype and therapeutic response. Its core lies in the synergistic action of multiple cell types and molecular mechanisms within the TME, including metabolic reprogramming, immune checkpoint regulation, cytokine release, and epigenetic modifications. This cross-talk mechanism poses a challenge for treating complex diseases but also provides a theoretical basis for developing multi-target combination therapies. Therefore, designing a small molecule Hes1 inhibitor represents a potential therapeutic approach for cancer. However, further research is needed to translate these strategies into clinical applications. Therapy targeting Hes1 may also be combined with personalized treatment. In the future, the heterogeneous functions of Hes1 can be analyzed using single-cell multi-omics, dynamic imaging techniques, and artificial intelligence models, advancing the realization of personalized treatment plans.

Tumor chemotherapy and molecular targeted therapy resistance represent a critical bottleneck in clinical treatment, necessitating innovative intervention strategies to overcome drug resistance barriers. Hes1, as a core effector molecule in the Notch signaling pathway and a pivotal regulatory node in tumor drug resistance, plays a significant role in tumor resistance. For instance, studies have demonstrated that the transcriptional downregulation of Hes1 sensitizes LUAD patients to EGFR-TKI, and targeted blockade of this signal can reverse EGFR-TKI resistance in LUAD (85). Furthermore, certain natural compounds can restore PP6 expression in KC cells and inhibit its ubiquitination by targeting Hes1 in psoriasis models (198), paving a new direction to overcome the off-target effects of traditional Notch inhibitors. Future research should further investigate the post-translational modifications of Hes1 and its relationship with tumor metabolic reprogramming, utilizing single-cell sequencing and organoid models to untangle its dynamic regulatory mechanisms. Additionally, it should explore how Hes1 precisely regulates the functional states of different types of immune cells and their spatial distribution within the TME, and whether targeting Hes1 (e.g., with combination inhibitors) can reshape the immunosuppressive microenvironment and subsequently enhance the anti-tumor efficacy of existing immunotherapies and overcome immune resistance. Although the expression level of Hes1 is significantly correlated with the prognosis of patients with malignant tumors, its value as an independent biomarker still requires validation through large samples.

In summary, the role of Hes1 in tumors not only provides abundant intervention targets but also demands that future research integrates multi-omics data or artificial intelligence prediction models. Particularly, there is a need for integrated studies on Hes1’s functions in both the intrinsic mechanisms of tumor cells and the regulation of the extrinsic immune microenvironment. This will facilitate the translation of Hes1-targeted therapy from the laboratory to the clinic, providing a solid evidentiary foundation for the development of innovative diagnostic and targeted therapeutic interventions for tumors, including immunotherapy.
